# Temporal lobe connects regression and macrocephaly to autism spectrum disorders

**DOI:** 10.1007/s00787-015-0746-9

**Published:** 2015-07-30

**Authors:** Giulia Valvo, Sara Baldini, Alessandra Retico, Giuseppe Rossi, Raffaella Tancredi, Anna Rita Ferrari, Sara Calderoni, Fabio Apicella, Filippo Muratori, Filippo Maria Santorelli, Federico Sicca

**Affiliations:** Department of Developmental Neuroscience, IRCCS Stella Maris Foundation, Via dei Giacinti 2 - Calambrone, 56128 Pisa, Italy; National Institute for Nuclear Physics (INFN), Pisa, Italy; Unit of Epidemiology and Biostatistics, Institute of Clinical Physiology, National Research Council (CNR), Pisa, Italy

**Keywords:** Autism, EEG, Regression, Macrocephaly, Temporal lobe

## Abstract

Interictal electroencephalogram (EEG) abnormalities are frequently associated with autism spectrum disorders (ASD), although their relationship with the clinical features of ASD, particularly the regressive onset, remains controversial. The aim of this study was to investigate whether the characteristics of interictal EEG abnormalities might help to distinguish and predict definite phenotypes within the heterogeneity of ASD. We reviewed the awake and sleep interictal EEGs of 220 individuals with idiopathic ASD, either with or without a history of seizures. EEG findings were analyzed with respect to a set of clinical variables to explore significant associations. A brain morphometry study was also carried out on a subgroup of patients. EEG abnormalities were seen in 154/220 individuals (70 %) and were mostly focal (*p* < 0.01) with an anterior localization (*p* < 0.001). They were detected more frequently during sleep (*p* < 0.01), and were associated with a regressive onset of ASD (*p* < 0.05), particularly in individuals with focal temporal localization (*p* < 0.05). This association was also stronger in regressive patients with concurrent macrocephaly, together with a relative volumetric reduction of the right temporal cortex (*p* < 0.05). Indeed, concurrence of temporal EEG abnormalities, regression and macrocephaly might possibly define a distinct endophenotype of ASD. EEG-based endophenotypes could be useful to untangle the complexity of ASD, helping to establish anatomic or pathophysiologic subtypes of the disorder.

**Electronic supplementary material:**

The online version of this article (doi:10.1007/s00787-015-0746-9) contains supplementary material, which is available to authorized users.

## Introduction

Electroencephalograms (EEG) abnormalities or seizures are highly comorbid with autism spectrum disorders (ASD) [1–4], possibly defining a distinct pathophysiological subgroup (Epileptiform-ASD Phenotype). The pooled prevalence of seizures in ASD ranges from 8 to 24 % [5–7], and up to 60–75 % of ASD patients are reported to display EEG abnormalities [8–12]. Several studies have recently focused on the relationship between seizures, EEG abnormalities, and clinical features of ASD, although they have not led to unifying results [9, 10, 13–16]. For example, some studies report a higher rate of EEG abnormalities in individuals with ASD and severe intellectual disability (ID) [17] or lower IQ [14], while others have found that only seizures are associated with disordered cognitive functioning in ASD [13, 18]. Similarly, behavior disorders (hyperactivity and irritability) have been linked to seizures but not EEG abnormalities in ASD [19, 20], whereas others have suggested that aggressiveness and stereotypes are associated with a high incidence of epileptiform abnormalities, but not with the presence of clinical seizures [16]. Also, our recent survey of overgrowth in ASD children has shown that tall stature may influence susceptibility to EEG abnormalities or late epilepsy and, when concurring with macrocephaly, predisposes to early onset seizures [21]. Indeed, disturbances of somatic growth regulation seem to distinguish subgroups of individuals with ASD [22–25]. One of the most highly debated issues, however, remains the relationship between epilepsy (or EEG abnormalities) and a regressive onset of ASD. Although this potential link has been pointed out by several years [26], questions remain, leaving this issue largely unresolved [9, 13].

In this explorative study, we have investigated the hypothesis that the occurrence of EEG features, and their type and localization, might help in distinguishing diverse clinical subgroups of ASD. To this aim, EEG findings were analyzed with respect to a large set of clinical variables, to explore significant associations within the wide clinical heterogeneity of the disorder.

## Methods

We reviewed the awake and sleep interictal EEGs of 220 individuals with idiopathic ASD, either with or without a history of seizures. The sample, partially overlapping with that of our previous work [21], included patients who underwent EEG recordings from January 2010 to December 2013 at our Institution (IRCCS Stella Maris, Pisa). Indications for the EEG study were a history of seizures in 58/220 patients (26.4 %), previous EEG abnormalities in 21/220 (9.5 %), regressive onset of ASD in 37.3 % (82/220), or other clinical reasons (familial history for epilepsy, EEG assessment prior to the introduction of antipsychotic medications) in 6/220 patients (2.7 %). EEG was done for purely research purposes in 53/220 patients (24.1 %). Patients with non-idiopathic forms of autism-epilepsy, such as children with congenital or acquired cerebral lesions or known genetic syndromes, as well as those who did not reach the sleep state during the EEG recording, were not included in the study. Video-EEG-polygraphic recordings had been digitally acquired, positioning the electrodes according to the 10–20 International System, and were visually inspected by two independent investigators (GV, FS). When EEG evaluations were discordant, we obtained a third opinion from another clinical expert (ARF). We defined two types of interictal EEG abnormalities: (1) paroxysms (spikes, sharp waves, spike and wave complexes), that could be focal or multifocal\diffuse, and (2) focal dysrhythmia or slowing [27]. Focal abnormalities (paroxysms or slowing) were also classified according to their site predominance in anterior (Fp2, F4, C4, Fp1, F3, C3, Fz, Cz), posterior (P4, O2, P3, O1, Pz, Oz), and temporal (F8, T4, T6, F7, T3, T5) brain regions, and as left-sided, right-sided or bilateral. We also classified the EEG abnormalities according to their occurrence only on awake or sleep states, or both.

The occurrence and site of EEG abnormalities were analyzed with respect to a set of clinical variables: age, gender, ASD diagnosis [Autism, Pervasive Developmental Disorder Not Otherwise Specified (PDDNOS), Asperger’s Syndrome], regressive versus non-regressive onset of ASD, presence of seizures and type of seizures (focal, generalized, spasms), level of cognitive and language development, presence of behavioral problems, and auxological parameters.

Despite that the Diagnostic and Statistical Manual of Mental Disorders, Fifth Edition (DSM-5, APA, 2013) no longer uses PDD-NOS or Asperger’s syndrome—since all subcategories are now included under the common definition of autism spectrum disorder—this study started prior to its publication, so we referred to the criteria for Pervasive Developmental Disorders of the DSM-IV-TR. Diagnoses were also corroborated in most cases (173/220; 78.6 %), with the gold-standard Autism Diagnostic Observation Schedule-Generic (ADOS-G) [28] administered by a psychologist certificated for research practice (FA). Developmental regression, defined as an abrupt or gradual loss of previously acquired language skills, developmental milestones, and social reciprocity [29, 30], was assessed through careful clinical interviews with the caregiver and reviews of previous data, and labeled as present or absent. The interviews were guided by the clinician in order to distinguish a true loss of skills from a developmental plateau or slowing, or transient fluctuations in behavior over brief periods. When information on regression was uncertain or questionable, we used a conservative approach and classified patients as non-regressive. Seizures, when present, were classified according to the classifications of the International League Against Epilepsy (ILAE) (1981, 1989) and the subsequent Report of the ILAE Commission on Classification and Terminology (2005–2009) [31]. Cognitive development was assessed through standardized tools [Griffiths Mental Development Scale Extended-Revised (GMDS-ER), Leiter International Performance Scale-Revised (LIPS-R), Wechsler Preschool and Primary School Intelligence Scale-III (WPPSI-III), and Wechsler Intelligence Scale for Children-IV (WISC-IV)] in 157/220 (71.4 %) individuals. When a standardized evaluation was not applicable, the cognitive level was estimated through in-depth multidisciplinary clinical observations. According to their cognitive development, patients were defined with normal-to-borderline, mild-to-moderate delay, or severe delay. Expressive language development, assessed through clinical observation, was classified as absent, delayed, or normal. Finally, height (H) and head circumference (HC) were measured by calibrated scales and their values plotted on standard growth charts for Ref. [32, 33]. Macrocephaly was defined as HC over the 97th percentile.

A brain morphometry study was carried out in a subgroup of patients with temporal EEG abnormalities, with either regression or macrocephaly or both, whose digital brain MRI data had been acquired at our site. The standard MRI protocol included a whole-brain fast spoiled gradient recalled acquisition in the steady-state T1-weighted series (FSPGR), collected in the axial plane yielding contiguous 1.1 mm axial slices, with an in-plane resolution of 1.1 × 1.1 mm^2^. The brain morphometric study of this sample has been conducted according to two different approaches: the voxel-based morphometry (VBM) analysis [34], and the statistical comparison among the global volumes of parceled regions of interest (ROIs) [35]. For both analyses, the T1-weighted MR images were processed with the statistical parametric mapping (SPM) (http://www.fil.ion.ucl.ac.uk/spm) software package SPM8 (Wellcome Department of Imaging Neuroscience, London, UK) as follows: (1) SPM segmentation of brain tissues, i.e., grey matter (GM), white matter (WM) and cerebrospinal fluid (CSF), using the New Segment toolbox; (2) the diffeomorphic anatomical registration using exponentiated lie algebra (DARTEL) algorithm was implemented to obtain a population-based brain template [36]; (3) affine transformation of the DARTEL template to the Montreal Neurological Institute (MNI) reference space and consequent diffeomorphic warping of the segmented brain tissues into MNI; (4) standard smoothing with isotropic Gaussian kernel (*s* = 10 mm), including the modulation operation [37], to make the final statistics reflecting the local volume differences in tissue segments. Once this preprocessing was completed, the smoothed, modulated and normalized images (warped in the MNI space) were used for both the VBM analysis and ROI parcellation followed by the statistical analyses. To investigate volumetric differences at the ROI level, the volumes of lobes (frontal, parietal, limbic, occipital, temporal) and other structures (insula, caudate, putamen, cerebellum and brainstem) were extracted from each subject according to the parcellation defined by the LONI Probabilistic Brain Atlas (http://www.loni.ucla.edu.Atlases/LPBA40) [35]. The ROIs of the LONI Probabilistic Brain Atlas were co-registered to the MR images and applied as masks to the segmented GM volumes of our data sample.

### Data analyses

Statistical analyses were conducted using the IBM© SPSS© Statistics software version 20 (Armonk, New York, United States). For continuous variables, including the ROI volumes, we performed two-tailed *t* tests, two-way analysis of variance (ANOVA), GLM Univariate analysis, and post hoc multiple comparisons using the Bonferroni correction. The option “exclude cases analysis by analysis” was chosen to manage missing data. For categorical variables, we used the Chi squared test and correspondence analysis (CA) to decompose the significant Chi squared and reduce variables dimensions. Significance was set at *p* < 0.05. Asterisks in the tables and figures indicate statistically significant results.

In the VBM analysis, to detect between-group differences, the segmented GM images of each group of subjects were entered into a voxel-wise two-sample *t*-test analysis in SPM8. We used an absolute threshold mask of 0.2 on GM to avoid possible edge effects around the border between GM and WM. The total GM volume was entered as covariate in the statistical analysis to take into account the subjects’ brain size variability. We set significance at *p* < 0.05 and used the family-wise error rate (FWE) correction for multiple comparisons.

## Results

**Fig. 1:**
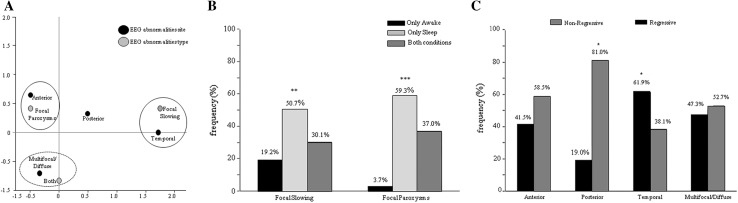
**a** Biplot for cross-tabulation of type of interictal EEG abnormalities by site: correspondence analysis at a two-dimensional solution showed that the focal paroxysms were associated with the anterior localization (*closed circle*), and the focal slowing with the temporal site (*closed circle*). The combination of both paroxysms and focal slowing was associated with multifocal/diffuse abnormalities (*dashed circle*). **b** Interictal EEG abnormalities were detected more frequently during sleep (**p ≤ 0.01, ***p ≤ 0.001). **c** The regressive onset of ASD was significantly associated with a temporal localization of interictal EEG abnormalities, whereas non-regressive individuals displayed mostly posterior abnormalities (*p ≤ 0.05)

The sample consisted of 220 patients [185 boys (M; 84.1 %) and 35 girls (F; 15.9 %)], aged 2.0–20.8 years with a mean age of 7.0 (±3.8 standard deviation, SD). Among them, 71 (32.3 %) received a diagnosis of Autism, while 146 (66.3 %) were classified as PDDNOS. Only three individuals (1.4 %) were diagnosed as having Asperger’s Syndrome. Interictal EEG abnormalities were detected in 154/220 individuals (70 %) and were mostly represented by paroxysms (81/154; 52.6 %), while focal slowing was recorded in 22/154 (14.3 %), and a combination of both types of EEG abnormalities was found in 51/154 patients (33.1 %) (*χ*^2^ = 33.909, *df* = 2, *p* = 0.001, *ɸc* = 0.332; Table 1). Taken together, EEG abnormalities were mostly focal (95 patients, 61.7 %), while 38.3 % (*n* = 59) of affected individuals displayed multifocal/diffuse abnormalities (*χ*^2^ = 8.416, *df* = 1, *p* = 0.004, *ɸc* = 0.234; Table 1). Also, focal abnormalities had an anterior localization in most cases (53/95; 55.8 %), whereas an equal amount of patients (22.1 %; *n* = 21) displayed either posterior or temporal abnormalities (*χ*^2^ = 21.558, *df* = 2, *p* < 0.001, *ɸc* = 0.337; Table 1). Anterior interictal EEG abnormalities were mainly bilateral or left-sided (*χ*^2^ = 7.396, *df* = 2, *p* = 0.025, *ɸc* = 0.264), while there was no prevalent side on posterior and temporal areas. Whilst interictal EEG abnormalities were mainly paroxysms on anterior regions, and focal slow activity over the temporal sites, we did not find differences in the type of abnormalities (paroxysms vs. focal slowing) over the posterior regions; when abnormalities had a multifocal/diffuse localization, they mostly presented with both paroxysms and focal slowing (*χ*^2^ = 66.964, *df* = 6, *p* < 0.001, *ɸc* = 0.659; see Table S1, Table S2 in Online Resource 1, and Figure 1a). Globally, EEG abnormalities, either focal slowing or paroxysms, were detected more frequently during sleep (focal slowing: *χ*^2^ = 11.205, *df* = 2, *p* = 0.004, *ɸc* = 0.277; paroxysms: *χ*^2^ = 63.333, *df* = 2, *p* < 0.001, *ɸc* = 0.484) (Fig. 1b).

**Table 1:** Characterization of interictal EEG abnormalities

	Sample size	Test	Effect size
Type***	154		
Paroxysms	81 (52.6 %)	*χ* ^2^ = 33.909	*ɸc* = 0.332
Focal slowing	22 (14.3 %)		
Both	51 (33.1 %)		
Localization**	154		
Focal	95 (61.7 %)	*χ* ^2^ = 8.416	*ɸc* = 0.234
Multifocal/Diffuse	59 (38.3 %)		
Site***	95		
Anterior	53 (55.8 %)	*χ* ^2^ = 21.558	*ɸc* = 0.337
Posterior	21 (22.1 %)		
Temporal	21 (22.1 %)		

*χ* ^2^ Pearson Chi squared test; *ɸc* Cramer’s phi coefficient

EEG abnormalities were mostly represented by paroxysms (***), with mainly focal (**), and anterior (***) localization

** *p* ≤ 0.01; *** *p* ≤ 0.001

A history of seizures was reported in 58/220 (26.4 %) individuals in the whole sample and all patients with epilepsy displayed EEG abnormalities. Among patients without seizures (162/220 [73.6 %]), EEG abnormalities were instead detected in 96 individuals (59.2 %). No differences in the localization of EEG abnormalities emerged between patients with and without seizures. Instead, patients with seizures significantly showed the concurrence of both type of interictal EEG abnormalities, namely paroxysms and focal slowing (*χ*^2^ = 12.133, *df* = 2, *p* = 0.002, *ɸc* = 0.281) (see Table S3 in Online Resource 1). EEG characteristics (localization, type and site), however, did not correlate with epilepsy features (age of onset and type of seizures). Also, the comparison of most phenotype features (age, gender, ASD diagnosis, language and cognitive development, regression, presence of behavioral problems and auxological parameters) between the two groups showed no significant differences even when we excluded patients with focal slowing, except for the mean age at last clinical assessment, which was higher in individuals with seizures, probably due to the presence of subjects with late onset epilepsy (Table S4 and S5).

**Fig. 2:**
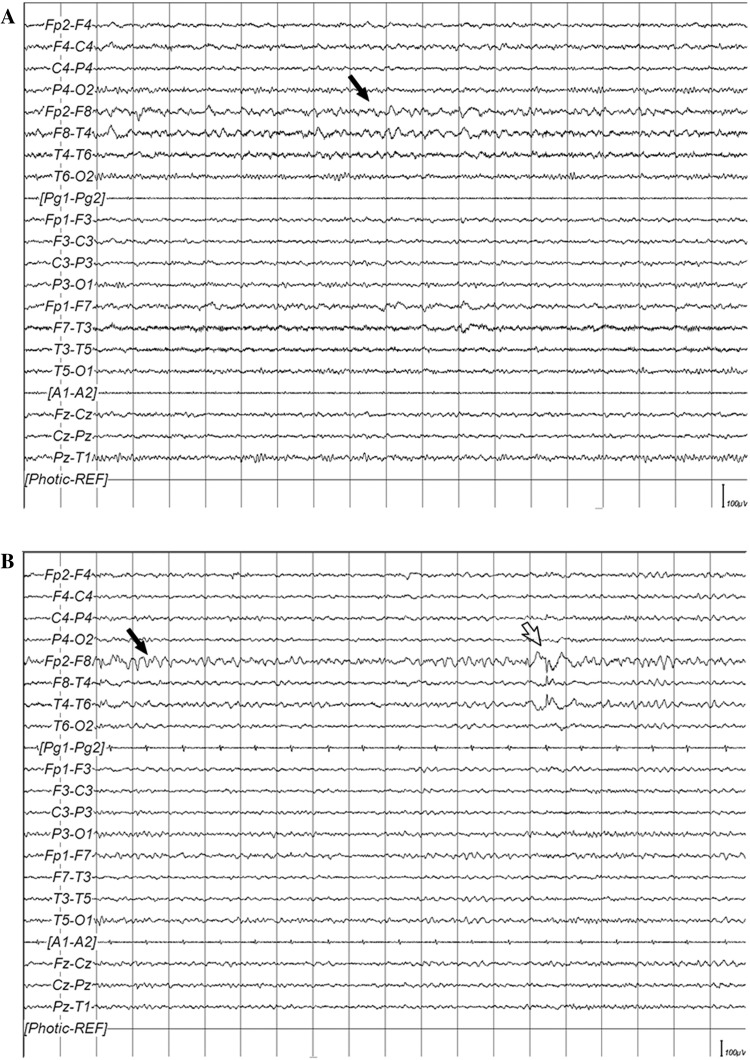
**a** Focal slowing (*black arrow*) over the right temporal region, in the awake state, in a 14-year-old boy. **b** Focal slowing (*black arrow*) and paroxysms (*white arrow*) over the right temporal region, during drowsiness, in a 15-year-old boy

The same phenotype features were also analyzed in children with EEG abnormalities (ASD-EEG), regardless of the presence of clinical seizures, comparing to children with normal EEG (ASD “simplex”). We found no significant differences, with the exception of a regressive onset of the disorder, which was clearly associated with the ASD-EEG group (*χ*^2^ = 5.338, *df* = 1, *p* = 0.021, *ɸc* = 0.158). Indeed, among 82 “regressive” patients (38.3 % of 214 cases; this information was not available in six), 65 (79.3 %) displayed an abnormal EEG (see Table S6 in Online Resource 1). The rate of regression in the ASD-EEG group, however, was not related to the history of clinical seizures (*χ*^2^ = 0.549, *df* = 1, *p* = 0.459, *ɸc* = 0.06). We found, moreover, that the regressive onset was also associated with the site of EEG abnormalities. By comparing the prevalent site (anterior, posterior, temporal, multifocal/diffuse) of EEG abnormalities in patients with and without regression, we found a significant association between a regressive onset of ASD with the temporal localization of EEG findings, and between a non-regressive onset with the posterior localization of EEG abnormalities (*χ*^2^ = 8.413, *df* = 3, *p* = 0.038, *ɸc* = 0.237) (Fig. 1c), regardless of whether EEG abnormalities were left or right-sided (temporal, *p* = 0.949; posterior, *p* = 1.000), or if there were paroxysms or focal slow activity (temporal, *p* = 0.298; posterior, *p* = 0.372). Instead, no associations emerged between the site or lateralization of EEG abnormalities and age, gender, type of ASD diagnosis, cognitive or language development, presence of behavioral problems and auxological parameters. These preliminary results made us wonder whether the regressive onset of ASD seen in the 38 % of our sample might eventually distinguish, within the heterogeneity of the Epileptiform-ASD phenotype, a different subtype of the disorder, where clinical and EEG features associate in a different way with respect to non-regressive individuals. To explore this hypothesis, we have stratified patients according to the regressive vs. non-regressive onset of ASD, and analyzed their clinical features with respect to the site of EEG abnormalities. We found that in the regressive-ASD subgroup, the presence of macrocephaly was significantly associated with temporal EEG abnormalities (*χ*^2^ = 10.649, *df* = 3, *p* = 0.014, *ɸc* = 0.414) (Table 2), suggesting the existence of a clinical endophenotype displaying regressive onset of ASD, temporal EEG abnormalities and macrocephaly. A representative example of the EEG abnormalities characterizing this subgroup is shown in Fig. 2.

**Table 2:** Site of interictal EEG abnormalities vs. macrocephaly in regressive and non-regressive individuals

Macrocephaly	Site				Effect size	Test
	Anterior	Posterior	Temporal	Multi/Diffuse		
Non-regressive						
No	21 (38.2 %)	11 (20 %)	5 (9.1 %)	18 (32.7 %)	*ɸc* = 0.062	*χ* ^2^ = 0.311
Yes	9 (33.3 %)	5 (18.5 %)	3 (11.1 %)	10 (37.1 %)		
Regressive						
No	17 (37 %)	1 (2.2 %)	6 (13 %)	22 (47.8 %)	*ɸc* = 0.414	*χ* ^2^ = 10.649
Yes^a^	3 (18.7 %)	2 (12.5 %)	7 (43.8 %)	4 (25 %)		

*χ* ^2^ Pearson Chi squared test; *ɸc* Cramer’s phi coefficient

* *p* ≤ 0.05

^a^In the regressive-onset subgroup, macrocephaly was significantly associated to the temporal localization of EEG abnormalities. Data of head circumference or ASD onset were missing in ten patients, therefore the analyses were performed on 144/154 individuals

**Fig. 3:**
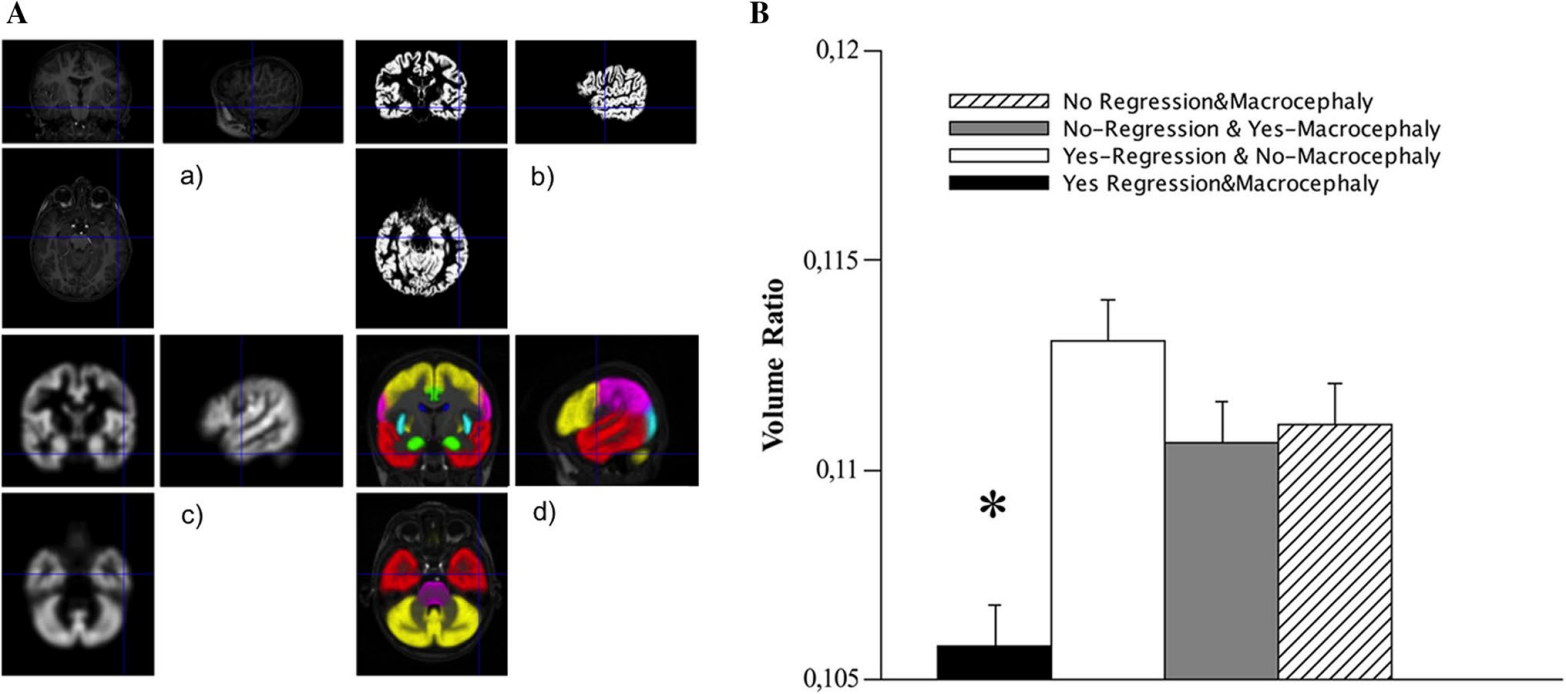
**a** Sketch of the analysis steps to estimate the regional volumes of brain structures: *a* original brain MRI of a representative subject; *b* segmented gray matter (GM); *c* modulated and smoothed GM, normalized to the Montreal Neurological Institute (MNI) reference space; *d* overlay of parceled ROIs according to the LONI Probabilistic Brain Atlas onto the MRI of the representative subject normalized to the MNI reference space; the following ROIs are visible: Frontal Lobes (*yellow*); Limbic Lobes (*green*); Occipital Lobes (*cyan*); Parietal Lobes (*magenta*); Temporal Lobes (*red*); Caudate (*blue*); Insular Cortex (*cyan*); Putamen (*yellow*); Brainstem (*magenta*); Cerebellum (*yellow*). **b** Volumetric differences of the right temporal lobe in individuals with temporal EEG abnormalities: patients with both macrocephaly and regression (*black histogram*) showed a significant reduction (*p ≤ 0.05) of the volume ratio (right temporal cortex/total *gray* matter). *Vertical bars* indicate standard error mean

Given that patients with temporal EEG discharges were the single subset displaying a significant association with phenotypic features, such as macrocephaly and regression, that might possibly define distinct subtypes of the disorder, we wondered whether they also had distinct brain anatomy profiles. Accordingly, we performed a morphometric study of brain MRI scans in 11/21 individuals with temporal EEG abnormalities [six of them (54.5 %) showing focal slowing, and five (45.5 %) both paroxysms and focal slowing]. MRI scans were not available in the remaining 10/21 children. We carried out both a VBM analysis and ROI volume comparisons, in subjects with regression (2/11; 9.2 %) or macrocephaly (3/11; 13.6 %), and also in those with both (3/11; 13.6 %), or neither (3/11; 13.6 %) feature. Subgroups were comparable with respect to gender, handedness, and mean age at the time of MRI study. No significant VBM differences were found between groups. Instead, the investigation of volumetric differences at the ROI level, by extracting regional volumes in each subject as sketched in Fig. 3a, revealed an interaction effect between the two independent variables, regression and macrocephaly (two-way ANOVA [GLM Univariate Analysis], *F* = 8.029, *df*_1_ = 1, *df*_2_ = 7, *p* = 0.025, partial eta squared = 0.534) only in the right temporal lobe (Fig. 3b and Table S7 in Online Resource 1). It appeared indeed that among individuals with temporal EEG abnormalities, those cases who had both macrocephaly and regression (3/11 patients) showed, as a unique significant finding, a reduced cortical volume in the right temporal lobe with respect to individuals with only regression but not macrocephaly (*t*-test, *t* = 3,654, *df* = 3, *p* = 0.035), and those with macrocephaly but not regression (*t*-test, *t* = 2.976, *df* = 4, *p* = 0.041).

## Discussion

In this study, we aimed at exploring which are the main types and locations of interictal EEG abnormalities in a sample of 220 individuals with idiopathic ASD, and to what extent EEG features may help to distinguish subgroups of the disorder.

The prevalence of EEG abnormalities or seizures in our sample agrees with rates already described in previous reports [8, 9, 12, 16], although the clinical indication for the EEG study due to a history of seizures (26.4 %) or previous EEG abnormalities (9.5 %), or regressive onset of ASD (37.3 %) might possibly represent a bias towards a higher percentage of patients with EEG abnormalities or epilepsy. However, this finding corroborates the observation that this association is more than the result of chance, but might be the consequence of shared pathogenic mechanisms that alter neuronal excitability leading to interictal EEG paroxysms or seizures, on one site, and the cognitive and behavioral dysfunctions characterizing ASD on the other [2]. We also confirmed that EEG abnormalities are mostly detected during sleep, in agreement with earlier data [9, 13, 16], suggesting that, in clinical practice, a single recording of wakefulness may considerably underestimate the presence of EEG abnormalities in patients with autism. Focal paroxysms, with a predominant anterior localization, were the main type of EEG feature in our sample, as already observed by others [14–16, 18, 38], whereas focal slowing was seen in a relatively small set of cases and appeared to have a predominant localization over the temporal regions. We have not a clear explanation for focal slowing in ASD. Previous research aimed at investigating the significance of EEG focal slowing, both isolated or associated with paroxysmal abnormalities, in children with epilepsy showed that it may witness the presence of structural lesions of the brain, to an extent at least equal to what happens with epileptiform abnormalities [39]. Our patients were all classified as having idiopathic ASD, and their neuroimaging, available in the large majority of children (65/73; 89 %) with focal EEG slowing, in the context of either focal or multifocal EEG patterns, confirmed the absence of overt lesions. This suggests that focal slowing in the Epileptiform-ASD Phenotype might outline a primarily functional defect of the brain, or possibly microstructural alterations involving localized cerebral regions, mainly the temporal area, which could eventually become evident only with post-processing analyses of structural brain MRI data.

Regardless of the type of abnormality, however, we found that EEG features were strongly indicative of a regressive onset of ASD, independently by the occurrence of seizures, although this association might be in part affected by a selection bias due to the 37 % of patients that underwent EEG based on a history of developmental regression.

It has been long debated in the epilepsy literature whether interictal epileptiform abnormalities, in the absence of clinical seizures, might affect cognitive development [40], or lead to behavioral regression [2, 26]. It is even harder to establish, in our sample, the causative relationships between abnormal EEG and the neurocognitive regression characterizing those children. We cannot assess, indeed, whether the EEG abnormalities had preceded or followed the onset of ASD, because all children underwent EEG recordings several months or years after onset of autistic regression. However, none of the patients displayed EEG findings consistent with the severity and typical features seen in the common forms of epileptic encephalopathies (such as epilepsy with continuous spikes and waves during sleep or West syndromes). Hence, it remains conceivable that a same noxa (or a set of etiologies), either genetic, or environmental or both, could exert a deleterious effect during a particularly vulnerable phase of brain development, accounting for both EEG abnormalities (and seizures\epilepsy) and the socio-communicative regression characterizing ASD.

The association of EEG abnormalities and autistic regression was particularly evident when EEG features were located over the temporal areas; conversely, posterior EEG abnormalities were associated with a non-regressive onset of the disorder (Fig. 1c). The finding of a higher proportion of EEG abnormalities in children with regressive ASD has already been reported [26], but correlations between localization of EEG changes and risk of regression are novel in the literature. Interestingly, in our sample, the association of regression with temporal EEG abnormalities was particularly strong in individuals with Epileptiform-ASD Phenotype and concurrent macrocephaly (Table 2). The correlation between regression and macrocephaly in ASD is still questioned, with literature data suggesting that abnormal cranial overgrowth is independent from the onset status [41, 42], and emerging recent findings suggesting an association between brain enlargement and regressive-onset of the disorder [43]. Our research supports the latter hypothesis, and proposes that the concurrence of temporal EEG abnormalities, regression and macrocephaly might define a distinct clinical\pathophysiologic subgroup of the Epileptiform-ASD Phenotype. This comorbid endophenotype was also characterized, in a subset of 11 individuals, by a distinct volumetric pattern at the brain morphometric study, which could not be explained by focal cortical abnormalities since brain MRI showed no lesions or malformations. Though we did not observe significant differences between regressive and non-regressive individuals, nor between macrocephalic and normocephalic patients as in previous research [44], we found a relative cortical volumetric reduction of the right temporal lobe in the subset of patients with temporal EEG abnormalities, macrocephaly and regression. Interestingly, the temporal lobe has a pivotal role in processing language and social stimuli [45], and is a crucial area of alterations detected with both structural MRI [46, 47] and post-mortem investigations in ASD [48, 49]. In particular, the right temporal region has been implicated in social perception, and in processing of face expressions, eye gaze, body movements, and emotional information, all core areas of impairment in ASD [50, 51].

Even if data in this work should be regarded cautiously, because of the relatively small number of patients with temporal EEG abnormalities, and even less with digital brain MRI analysed, and because of the low-to-moderate effect sizes that limit the strength of the statistical associations, our results indicate the temporal lobe as a possible target of the pathological neurodevelopmental processes underlying regression in autism, especially if combined with abnormal EEG. EEG-based endophenotypes could be useful to untangle the complexity of ASD, shedding light on possibly distinct anatomic or pathophysiologic subtypes of the disorder. The comorbid ASD endophenotype, in our study, characterized by temporal abnormalities, regression, and macrocephaly, might indeed have distinct disease mechanisms deserving further investigation with targeted clinical, neurophysiological and neuroimaging studies. It is tempting to imagine that reducing the clinical heterogeneity of ASD, by defining more homogeneous endophenotypes, could be used for more focused DNA testing, or for more straightforward correlation with the multitude of data emerging by second generation DNA sequencing methodologies.

## Electronic supplementary material

Below is the link to the electronic supplementary material.

Supplementary material 1 (PDF 238 kb). Supplementary Material may be found in the online version of this article. Online Resource 1: Table S1 Type of interictal EEG abnormalities according to their site. Table S2 Correspondence Analysis of Site of interictal EEG abnormalities vs. Type of EEG abnormalities. Table S3 Site and Type of interictal EEG abnormalities according to presence or absence of seizures. Table S4 Analysis of clinical variables in individuals with interictal EEG abnormalities, according to presence or absence of seizures. Table S5 Analysis of clinical variables in individuals with interictal EEG abnormalities (excluding those with only focal slowing), according to presence or absence of seizures. Table S6 Presence of interictal EEG abnormalities is related to regressive onset of ASD. Table S7 Volumetric analysis at the ROI level.
