# Supplementary material for: Temporal lobe connects regression and macrocephaly to autism spectrum disorders
Source: Eur Child Adolesc Psychiatry. 2015 Jul 30;25:421–9. doi: 10.1007/s00787-015-0746-9 (PMC4820486; doi:10.1007/s00787-015-0746-9)
Supplement: Supplementary file 1 — Supplementary material 1 (PDF 238 kb). Supplementary Material may be found in the online version of this article. Online Resource 1: Table S1 Type of interictal EEG abnormalities according to their site. Table S2 Correspondence Analysis of Site of interictal EEG abnormalities vs. Type of EEG abnormalities. Table S3 Site and Type of interictal EEG abnormalities according to presence or absence of seizures. Table S4 Analysis of clinical variables in individuals with interictal EEG abnormalities, according to presence or absence of seizures. Table S5 Analysis of clinical variables in individuals with interictal EEG abnormalities (excluding those with only focal slowing), according to presence or absence of seizures. Table S6 Presence of interictal EEG abnormalities is related to regressive onset of ASD. Table S7 Volumetric analysis at the ROI level. [file 787_2015_746_MOESM1_ESM.pdf]

European Child & Adolescent Psychiatry

Temporal Lobe connects Regression and Macrocephaly to Autism Spectrum Disorders

Giulia Valvo[1], Sara Baldini[1], Alessandra Retico[2], Giuseppe Rossi[3], Raffaella Tancredi[1], Anna Rita Ferrari[1], Sara Calderoni[1], Fabio Apicella[1], Filippo Muratori[1], Filippo Maria Santorelli[1], and Federico Sicca[1]

[1] Department of Developmental Neuroscience, IRCCS Stella Maris Foundation, Pisa, Italy

[2] National Institute for Nuclear Physics, Pisa Division, Italy

[3] Unit of Epidemiology and Biostatistics, Institute of Clinical Physiology, National Research Council (CNR), Pisa, Italy

Corresponding Author

Dr. Federico Sicca, Clinical Neurophysiology Laboratory - Department of Developmental Neuroscience

IRCCS Stella Maris Foundation - Via dei Giacinti, 2, Calambrone - 56128 Pisa – Italy,

Email: federico.sicca@fsm.unipi.it - Phone Number +39050886234, Fax Number +39050886247.

Table S1 Type of interictal EEG abnormalities according to their site

	Anterior n: 53	Posterior n:21	Temporal n:21	Multifocal/Diffuse n:59	Effect size	Test
**EEG Type (n) *** **						
Paroxysms (81)	42 (79,2%)	10 (47,6%)	2 (9,5%)	27 (45,8%)	ϕc=0.659	χ^2=66.964
Focal slowing (22)	3 (5,7%)	6 (28,6%)	12 (57,1%)	1 (1,7%)		
Both (51)	8 (15,1%)	5 (23,8%)	7 (33,3%)	31 (52,5%)		

n= number of patients, χ^2= Pearson chi-squared test; ϕc= Cramer's phi coefficient; *** p≤0.001. Focal paroxysms were prevalent on anterior areas, focal slowing on temporal regions, and both types of EEG abnormalities in multifocal/diffuse localization (***)

Table S2 Correspondence Analysis of Site of interictal EEG abnormalities vs. Type of EEG abnormalities

Summary

				**Proportion of Inertia**		**Confidence Singular Value**	
**Dimension**	**Inertia**	**Chi Square**	**Sig.**	Accounted for	Cumulative	Standard Deviation	Correlation
1	0.314			0.722	0.722	0.078	-0.008
2	0.121			0.278	1.000	0.074	
Total	0.435	66.964	0.000	1.000	1.000		

Overview Row Points

	**Score in Dimension**			**Contribution**				
				Of Point to Inertia of Dimension		Of Dimension to Inertia of Point		
**Site**	**1**	**2**	**Inertia**	**1**	**2**	**1**	**2**	**Total**
Anterior	-0.498	0.649	0.098	0.152	0.416	0.486	0.514	1.000
Posterior	0.490	0.336	0.024	0.058	0.044	0.774	0.226	1.000
Temporal	1.710	-0.006	0.223	0.712	0.000	1.000	0.000	1.000
Multifocal/ Diffuse	-0.336	-0.700	0.090	0.077	0.540	0.271	0.729	1.000
Active Total			0.435	1.000	1.000			

Overview Column Points

	**Score in Dimension**			**Contribution**				
				Of Point to Inertia of Dimension		Of Dimension to Inertia of Point		
**Type**	**1**	**2**	**Inertia**	**1**	**2**	**1**	**2**	**Total**
Paroxysms	-0.477	0.415	0.099	0.214	0.260	0.681	0.319	1.000
Focal slowing	1.756	0.416	0.255	0.786	0.071	0.966	0.034	1.000
Both	0.001	-0.838	0.081	0.000	0.669	0.000	1.000	1.000
Active Total			0.435	1.000	1.000			

CA (two dimensions): **1° Dimension** (72% of total inertia): **in row (Site of EEG abnormalities)**, Temporal has the higher contribution to inertia (71%, positive sign), followed by Anterior (15%, negative sign); **in column (Type of EEG abnormalities)**, Focal slowing has the higher contribution to inertia (79%, positive sign), followed by Paroxysms (21%, negative sign). **2° Dimension** (28% of total inertia): **in row (Site of EEG abnormalities)**, Multifocal/Diffuse has the higher contribution to inertia (54%, negative sign), followed by Anterior (41%, positive sign); **in column (Type of EEG abnormalities)**, Both has the higher contribution to inertia (67%, negative sign), followed by Paroxysms (26%, positive sign)

Table S3 Site and Type of interictal EEG abnormalities according to presence or absence of seizures

	**Seizures**	**No seizures**	**Effect size**	**Test**
**Site**				
Anterior	17 (29.3%)	36 (37.5%)	ϕc=0.103	χ^2=1.637
Posterior	10 (17.2%)	11 (11.5%)		
Temporal	8 (13.8%)	13 (13.5)		
Multifocal/Diffuse	23 (39.7%)	36 (37.5%)		
**Type ****				
Paroxysms	22 (37.9%)	59 (61.5%)	ϕc=0.281	χ^2=12.133
Focal slowing	7 (12.1%)	15 (15.6%)		
Both	29 (50%)	22 (22.9%)		

χ^2= Pearson chi-squared test; ϕc= Cramer's phi coefficient; ** $p \leq 0.01$.

Patients with seizures displayed a significant association with the concurrence of both types of EEG abnormalities (paroxysms and focal slowing)

Table S4 Analysis of clinical variables in individuals with interictal EEG abnormalities, according to presence or absence of seizures

	Seizures	No seizures	Effect size	Test
**Age (mean±SD; range)**	9.2±5.0; 2.2-20.8	6.9±3.2; 2.0-17.5	-	t=3.185
**Gender**				
Male	49 (84.5%)	77 (80.2%)	ϕc=0.054	χ^2=0.444
Female	9 (15.5%)	19 (19.8%)		
**ASD diagnosis**				
Autism	20 (34.5%)	29 (30.2%)	ϕc=0.192	χ^2=5.660
PDDNOS	35 (60.3%)	67 (69.8%)		
Asperger	3 (5.2%)	0		
**Language Development**				
Normal	14 (24.1%)	19 (19.8%)	ϕc=0.143	χ^2=3.133
Delayed	23 (39.7%)	52 (54.2%)		
Absent	21 (36.2%)	25 (26%)		
**Cognitive Development**				
Normal-Borderline	18 (34%)	37 (40.2%)	ϕc=0.109	χ^2=1.709
Mild-Moderate Delay	19 (35.8%)	36 (39.1%)		
Severe Delay	16 (30.2%)	19 (20.7%)		
**Regression**				
Yes	26 (47.3%)	39 (41.1%)	ϕc=0.06	χ^2=0.549
No	29 (52.7%)	56 (58.9%)		
**Macrocephaly**				
Yes	20 (36.4%)	24 (25.8%)	ϕc=0.112	χ^2=1.844
No	35 (63.6%)	69 (74.2%)		
**Tall stature**				
Yes	6 (10.9%)	12 (13.3%)	ϕc=0.036	χ^2=0.185
No	49 (89.1%)	78 (86.7%)		
**Behavioral problems**				
Yes	30 (54.5%)	51 (53.7%)	ϕc=0.008	χ^2=0.010
No	25 (45.5%)	44 (46.3%)		

t=t-test; χ^2= Pearson chi-squared test; ϕc= Cramer's phi coefficient ; ** p≤0.01 Mean age was higher in individuals with seizures

Table S5 Analysis of clinical variables in individuals with interictal EEG abnormalities (excluding those with only focal slowing), according to presence or absence of seizures

	Seizures	No seizures	Effect size	Test
**Age (mean±SD; range)**	8.9±4.7; 2.2-19.4	6.7±3.3; 2.0-17.5	-	t=2.860
**Gender**				
Male	43 (84.3%)	64 (79%)	ϕc=0.066	χ^2=0.573
Female	8 (15.7%)	17 (21%)		
**ASD diagnosis**				
Autism	17 (33.3%)	23 (28.4%)	ϕc=0.205	χ^2=5.560
PDDNOS	31 (60.8%)	58 (71.6%)		
Asperger	3 (5.9%)	0		
**Language Development**				
Normal	13 (25.5%)	17 (21%)	ϕc=0.104	χ^2=1.430
Delayed	21 (41.2%)	42 (51.9%)		
Absent	17 (33.3%)	22 (27.1%)		
**Cognitive Development**				
Normal-Borderline	17 (37%)	33 (42.3%)	ϕc=0.090	χ^2=0.994
Mild-Moderate Delay	16 (34.8%)	29 (37.2%)		
Severe Delay	13 (28.2%)	16 (20.5%)		
**Regression**				
Yes	22 (45.8%)	31 (38.8%)	ϕc=0.070	χ^2=0.620
No	26 (54.2%)	49 (61.2%)		
**Macrocephaly**				
Yes	16 (32.7%)	19 (24.4%)	ϕc=0.090	χ^2=1.037
No	33 (67.3%)	59 (75.6%)		
**Tall stature**				
Yes	6 (12.2%)	9 (12%)	ϕc=0.004	χ^2=0.002
No	43 (87.8%)	66 (88%)		
**Behavioral problems**				
Yes	26 (54.2%)	43 (53.8%)	ϕc=0.004	χ^2=0.002
No	22 (45.8%)	37 (46.2%)		

t=t-test; χ^2= Pearson chi-squared test; ϕc= Cramer's phi coefficient ; ** p≤0.01 Mean age was higher in individuals with seizures

Table S6 Presence of interictal EEG abnormalities is related to regressive onset of ASD

Onset vs EEG groups *	Regressive (n=82)	Non-Regressive (n=132)	Effect size	Test
ASD-EEG	65 (79.3%)	85 (64.4%)	ϕc=0.158	χ^2=5.338
ASD "simplex"	17 (20.7%)	47 (35.6%)		

χ^2= Pearson chi-squared test; ϕc= Cramer's phi coefficient; * $p \leq 0.05$.

Regressive onset of ASD was significantly associated to the ASD-EEG group (*)

Table S7 Volumetric analysis at the ROI level

ROI	Regression	Macrocephaly	Descriptive<br>Mean	Std. Deviation	GLM Univariate Analysis<br>F	p	Partial Eta Squared
**Left hemisphere**	No	No	0.40298733	0.004632004	0.001	0.974	0.000
		Yes	0.40917733	0.006943139			
	Yes	No	0.39993600	0.020363261			
		Yes	0.40649533	0.002804229			
**Right hemisphere**	No	No	0.40615733	0.003131560	0.025	0.878	0.004
		Yes	0.40544000	0.002320436			
	Yes	No	0.40304050	0.011253604			
		Yes	0.40115867	0.006762369			
**Left Frontal Lobe**	No	No	0.14557633	0.002787111	0.951	0.362	0.120
		Yes	0.14719867	0.002258127			
	Yes	No	0.13924050	0.000304763			
		Yes	0.14755133	0.009840529			
**Right Frontal Lobe**	No	No	0.14637867	0.000658002	3.104	0.121	0.307
		Yes	0.14503067	0.002396949			
	Yes	No	0.13635250	0.002317189			
		Yes	0.14436633	0.007550620			
**Left Parietal Lobe**	No	No	0.08288400	0.001098603	0.019	0.895	0.003
		Yes	0.08629833	0.003321065			
	Yes	No	0.08332900	0.016085265			
		Yes	0.08561967	0.003823161			
**Right Parietal Lobe**	No	No	0.08176333	0.001452419	0.146	0.714	0.020
		Yes	0.08411700	0.004379908			
	Yes	No	0.08453450	0.011609986			
		Yes	0.08441100	0.003113454			
**Left Limbic Lobe**	No	No	0.02302067	0.000375758	1.499	0.260	0.176
		Yes	0.02499633	0.002440135			
	Yes	No	0.02335750	0.000753069			
		Yes	0.02311200	,001140100			
**Right Limbic Lobe**	No	No	0.02199933	0.000826109	1.193	0.311	0.146
		Yes	0.02400867	0.002270815			
	Yes	No	0.02222500	0.000151321			
		Yes	0.02234967	0.001047550			
**Left Occipital Lobe**	No	No	0.04144700	0.003523421	0.093	0.769	0.013
		Yes	0.03904033	0.001648448			
	Yes	No	0.04523750	0.004851460			
		Yes	0.04168467	0.002468380			
**Right Occipital Lobe**	No	No	0.04493033	0.002047379	0.099	0.762	0.014
		Yes	0.04163200	0.001645102			
	Yes	No	0.04684700	0.001374616			
		Yes	0.04423467	0.001808869			

**Left Temporal Lobe**	No	No	0.11005900	0.003985245	0.271	0.618	0.037
		Yes	0.11164467	0.001657740			
	Yes	No	0.10877150	0.001631295			
		Yes	0.10852733	0.002971575			
**Right Temporal Lobe ***	No	No	0.11108600	0.002320124	8.029	**0.025**	0.534
		Yes	0.11065233	0.001050177			
	Yes	No	0.11308200	0.000738219			
		Yes *	0.10579700	0.002623600			
**Left Insula**	No	No	0.01210700	0.000565833	0.413	0.541	0.056
		Yes	0.01258767	0.000773793			
	Yes	No	0.01233550	0.000482954			
		Yes	0.01233867	0.000503794			
**Right Insula**	No	No	0.01128333	0.000730871	1.858	0.215	0.210
		Yes	0.01209967	0.000952960			
	Yes	No	0.01153050	0.000020506			
		Yes	0.01118700	0.000496617			
**Left Caudato**	No	No	0.00514067	0.000112647	0.194	0.673	0.027
		Yes	0.00530433	0.000145225			
	Yes	No	0.00516650	0.000325976			
		Yes	0.00551733	0.000578122			
**Right Caudato**	No	No	0.00494567	0.000111267	0.030	0.867	0.004
		Yes	0.00505733	0.000154649			
	Yes	No	0.00515500	0.000455377			
		Yes	0.00534800	0.000611532			
**Left Putamen**	No	No	0.00514000	0.000735268	4.972	0.061	0.415
		Yes	0.00569033	0.000067885			
	Yes	No	0.00622200	0.000441235			
		Yes	0.00543000	0.000450551			
**Right Putamen**	No	No	0.00514667	0.000793739	3.413	0.107	0.328
		Yes	0.00571267	0.000275558			
	Yes	No	0.00606600	0.000384666			
		Yes	0.00547833	0.000360673			
**Cerebellum**	No	No	0.13790900	0.006639766	0.061	0.812	0.009
		Yes	0.13004900	0.008946755			
	Yes	No	0.14081700	0.032949762			
		Yes	0.13752367	0.011448932			
**Brainstem**	No	No	0.00918300	0.000294250	0.023	0.883	0.003
		Yes	0.00888300	0.000626266			
	Yes	No	0.00973150	0.000734684			
		Yes	0.00952300	0.000296565			

ROI= region of interest. Descriptive: mean and SD of the volume ratios (ROI cortex /total gray matter). GLM Univariate Analysis: F=two-way ANOVA; p= significance; Partial Eta Squared=effect size; * p≤ 0.05 (significant reduction of the volume ratio at the right temporal lobe in patients with both macrocephaly and regression)
